# Cognitive impairments in patients with treatment resistant epilepsy: Complex rehabilitation in university clinic

**DOI:** 10.1192/j.eurpsy.2021.680

**Published:** 2021-08-13

**Authors:** V. Korostiy, A. Maltsev, I. Blazhina

**Affiliations:** 1 Psychiatry, Narcology, Medical Psychology And Social Work, Kharkiv National Medical University, Kharkiv, Ukraine; 2 University Clinic, Kharkiv National Medical University, Kharkiv, Ukraine; 3 Department Of Nervous Diseases Psychiatry And Medical Psychology, Bucovinian State Medical University, Chernivtsi, Ukraine

**Keywords:** cognitive impairment, Epilepsy, university clinic, Rehabilitation

## Abstract

**Introduction:**

Сognitive deficit significantly affects the quality of life of patients. Aims of research was detection of cognitive impairments of varying degrees in epilepsy, and as well as studying the results of complex treatment in conditions of University clinic, physical and psychological rehabilitation, cognitive training and VNS included.

**Objectives:**

We studied the features of clinical and psychopathological manifestations of cognitive impairments in patients suffering from epilepsy.

**Methods:**

The study was attended by 100 patients (35 men and 65 women) who were inpatient care. The following psychodiagnostic techniques were used: the Toronto Cognitive Assessment TorCA, the test of 10 words of Luria, the MOCA test, the Münsterberg test, the quality of life scale, the Hamilton scale of depression and anxiety.

**Results:**

MCI was observed in 88 % patients, dementia in 12 % (50 % - mild dementia, in 24 % - moderate dementia and in 16% - severe dementia). We used non-pharmacological rehabilitation methods for correction of cognitive impairment in epileptic patients with MCI and mild dementia during 3 mounth.. Improving of cognitive function was observed in 48 % patients, stable level of cognitive function - in 36 %, progressing of cognitive imparment - in 16 % patiens with epilepsy.

**Conclusions:**

The results of the conducted research indicate the need for further study of the features of cognitive disorders in pharmacologically treatment resistant epilepsy and implementation of training aimed at improving cognitive function and preventing the progression of cognitive impairment in complex treatment of those patients.

